# Evaluation of electrophysiological characteristics and ventricular synchrony: An intrapatient‐controlled study during His‐Purkinje conduction system pacing versus right ventricular pacing

**DOI:** 10.1002/clc.23837

**Published:** 2022-05-03

**Authors:** Xueying Chen, Xiaolan Zhou, Yanan Wang, Qinchun Jin, Yufei Chen, Jingfeng Wang, Shengmei Qin, Jin Bai, Wei Wang, Yixiu Liang, Haiyan Chen, Yangang Su, Junbo Ge

**Affiliations:** ^1^ Department of Cardiology, National Clinical Research Center for Interventional Medicine, Shanghai Clinical Research Center for Interventional Medicine, Shanghai Institute of Cardiovascular Diseases Zhongshan Hospital of Fudan University Shanghai China; ^2^ Huashan Worldwide Medical Center, Huashan Hospital Fudan University Shanghai China; ^3^ Department of Echocardiology, Zhongshan Hospital Fudan University Shanghai China

**Keywords:** atrioventricular block, electromechanical synchronization, His bundle pacing, His‐Purkinje conduction system pacing, left bundle branch pacing

## Abstract

**Objectives to Background:**

To compare electromechanical ventricular synchrony when pacing from different sites, including right ventricular apex pacing (RVAP), right ventricular septum pacing (RVSP), His bundle pacing (HBP), left bundle branch pacing (LBBP), and RVSP during unipolar pacing from the ring electrode of LBBP lead (RVSP_ring_) in each patient and evaluate the correlations between electrophysiological characteristics and ventricular synchrony.

**Methods:**

Twenty patients with complete atrioventricular block indicated for dual‐chamber pacemaker implantation were included in the study. Unipolar pacing at different sites, including RVAP, RVSP, HBP, LBBP, and RVSP_ring_, was successively performed in each patient. The pacing characteristics and echocardiogram parameters were collected and compared among intrinsic rhythm and pacing at different sites.

**Results:**

Similar to HBP (114.84 ± 18.67 ms), narrower paced QRSd was found in LBBP (116.15 ± 11.60 ms) as compared to RVSP_ring_ (135.11 ± 13.68 ms), RVSP (141.65 ± 14.26 ms), and RVAP (160.15 ± 19.35 ms) (*p *< .001). LBBP showed comparable pacing parameters to RVAP or RVSP and was significantly better than HBP, with maintained cardiac function. TS‐12‐SD was significantly improved in LBBP (41.80 ± 20.97 ms) than RVAP (69.70 ± 32.42 ms, *p* = .003) and RVSP (63.30.56 ± 32.53 ms, *p* = .018) but similar to HBP (51.50 ± 25.67 ms, *p* = .283) or RVSP_ring_ (57.80 ± 25.65 ms, *p* = .198). Among these pacing strategies, negative values of interventricular mechanical delay (IVMD) were only identified in LBBP (−19.25 ± 18.43 ms), significantly different from RVAP (35.00 ± 30.72 ms), RVSP (22.85 ± 22.05 ms), HBP (5.20 ± 18.64 ms), and RVSP_ring_ (16.00 ± 26.76 ms (all *p* < .05). Using Pearson's analysis, Sti‐LVAT was positively correlated with QRS duration, IVMD, TS‐12‐SD, LVEDV, and LVESV, while a negative relationship could be observed for left ventricular ejection fraction.

**Conclusions:**

His‐Purkinje conduction system pacing (HPCSP) achieved better electrical and mechanical synchrony than conventional RV pacing. For interventricular synchrony, only LBBP initiated earlier LV activation than RV, in accordance with the right bundle branch block (RBBB) pattern of paced QRS during LBBP. Sti‐LVAT might be a good parameter correlating with LV systolic function and mechanical synchrony.

**Abbreviations**
AVB: atrioventricular block
EP: electrophysiology
HBP: His bundle pacing
HPCSP: His‐Purkinje conduction system pacing
IVCD: intraventricular conduction defect
IVMD: interventricular mechanical delay
LBBP: left bundle branch pacing
LVEF: left ventricular ejection fraction
LVEDV: left ventricular end‐diastolic volume
LVESV: left ventricular end‐systolic volume
RBBB: right bundle branch block
RVAP: right ventricular apex pacing
RVP: right ventricular pacing
RVSP: right ventricular septum pacing
RVSP_ring_
: RVSP during unipolar pacing from the ring electrode of LBBP lead
Sti‐LVAT: the stimulus to left ventricular activation time
TAPSE: tricuspid annular plane systolic excursion
TS‐12‐SD: standard deviation of the time‐to‐peak myocardial sustained velocity of 12 left ventricular segments

## 1 BACKGROUND

For decades, right ventricular pacing (RVP) has been established as a conventional pacing technique indicated for symptomatic bradycardia for its pacing efficacy and safety. However, RVP is obviously not a physiological pacing strategy and it could lead to interventricular and intraventricular electrical desynchrony, which might result in atrial fibrillation and heart failure,^1^, ^2^, ^3^ especially in ventricular pacing dependent patients.

His‐Purkinje conduction system pacing (HPCSP), His bundle pacing (HBP), and left bundle branch pacing (LBBP), have emerged as two physiological conduction system pacing strategies and have been demonstrated to achieve narrower QRS duration and better mechanical synchrony than RVP.^4^ Nowadays, HPCSP has been widely adopted from inside and outside the field.^5^ Recent studies have also demonstrated that HPCSP significantly improved heart function in heart failure patients with left bundle branch block (LBBB),^6^, ^7^, ^8^ and even better than BVP.^9^, ^10^ Theoretically, HBP is the most physiological pacing mode and it could achieve the same narrow QRS complex as the intrinsic and correct LBBB or right bundle branch block (RBBB) as well. However, pacing safety concerning high pacing threshold and low R wave amplitude of HBP limits its application in all pacing candidates, especially in infra‐Hisian block cases.^11^ While pacing captures the left conduction system more distal than His bundle, LBBP offers a low and stable threshold and high R wave amplitude comparable to RVP and its feasibility has been confirmed in large‐scale studies with mid‐long term follow‐up.^12^, ^13^, ^14^ LBBP activates the left ventricle earlier than the right ventricle through the left conduction system. Although paced QRS of LBBP is narrower than RVP from the literature, it shows an RBBB morphology that is different from the intrinsic. Consequently, it remains unknown that its RBBB shape would result in ventricular mechanical desynchrony as compared to HBP though previous case‐controlled studies have demonstrated its synchrony similar to HBP and superior to RVP.^15^, ^16^ However, the definitions of LBBP in these studies were not unified and the study populations were not ventricular pacing dependent. It would be more reliable to compare electromechanical synchrony at different pacing sites in each case in typically ventricular pacing dependent patients. Whereas, till now, direct comparisons of electromechanical effects in HPCSP and conventional RVP have not been well estimated. Additionally, an intrapatient analysis focusing on solely atrioventricular block (AVB) patients to balance individual differences has never been performed in previous research. Moreover, it is also unclear which electrophysiological value has the best correlations with ventricular synchrony and left ventricular function. Hence, the purpose of our present study is to draw meaningful comparisons between the electrophysiological and echocardiographic parameters during HPCSP and conventional RVP in AVB from an intrapatient analysis to further assess the relationship between electrical and mechanical synchrony.

## 2 METHODS

### 2.1 Study population

Twenty patients with complete AVB indicated for dual‐chamber pacemaker implantation were included in the study. Exclusion criteria included persistent atrial fibrillation, escape beat of intraventricular conduction defect (IVCD) morphology, and left ventricular ejection fraction (LVEF) < 50%. Written informed consent was obtained from all of the participants and the study was approved by the Institutional Review Board of Zhongshan Hospital, Fudan University, Shanghai, China.

### 2.2 Implantation procedure

**Figure 1:**
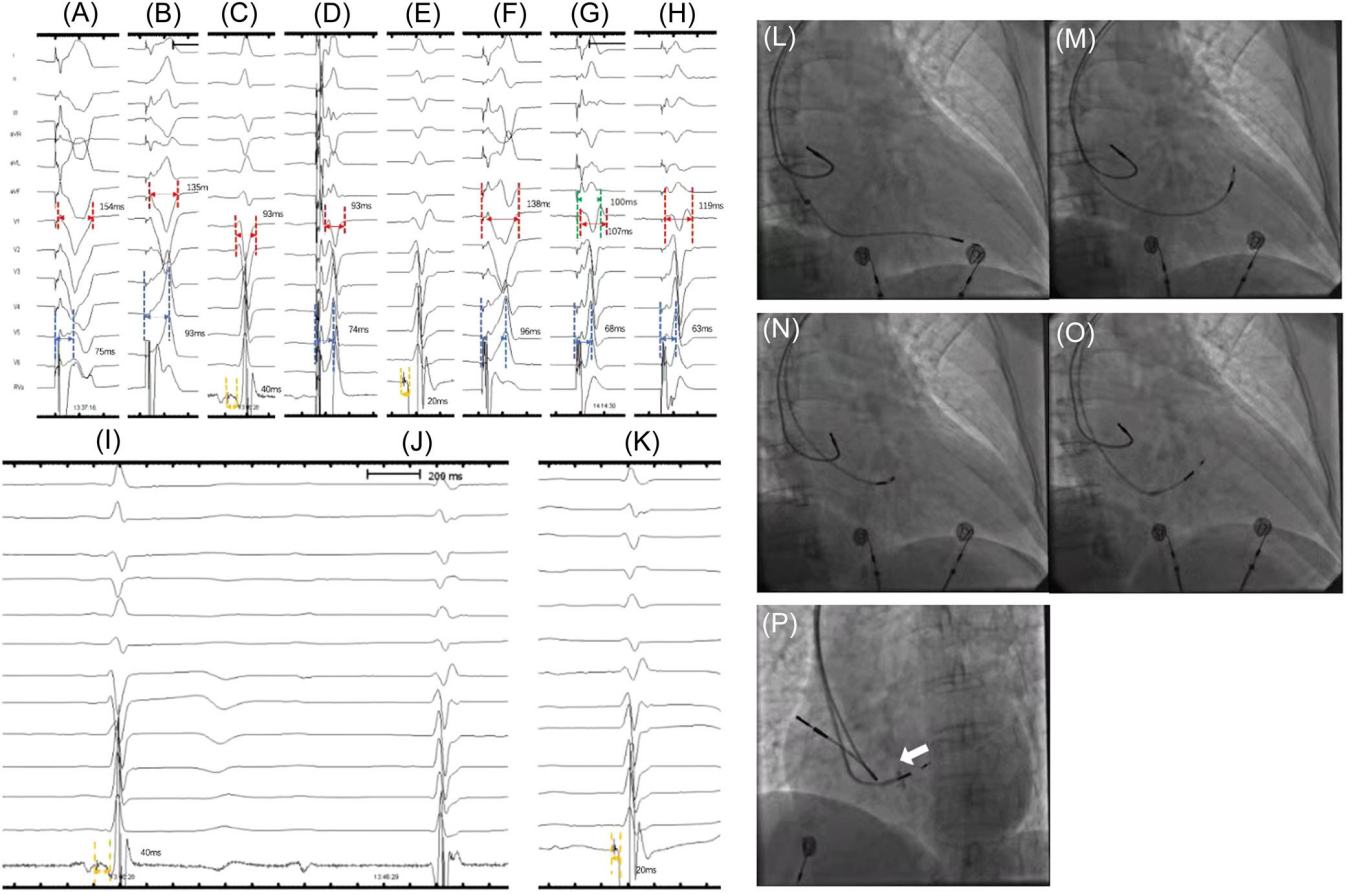
The 12‐lead ECG, EGM, and fluoroscopic images during pacing at different sites. (A) The 12‐lead ECG and the EGM recorded during RVAP: The paced QRSd was 154 ms while Sti‐LVAT was 75 ms. (B) The 12‐lead ECG and the EGM recorded during RVSP: The paced QRSd 135 ms was while Sti‐LVAT was 93 ms. (C) Po_His_ during the intrinsic rhythm with the potential to ventricle interval were 40 ms. (D) The 12‐lead ECG and the EGM recorded during HBP: The paced QRSd was 93 ms while Sti‐LVAT was 74 ms. (E) Po_LBB_ during the intrinsic rhythm with the potential to ventricle interval were 18 ms. (F) The 12‐lead ECG and the EGM recorded by unipolar pacing from the ring electrode of the LBBP lead (RVSP ring): The paced QRSd was 138 ms while Sti‐LVAT was 96 ms. (G) The 12‐lead ECG and the EGM recorded by the LBBP lead (Nonselective LBBP): The paced QRSd was 107 ms while Sti‐LVAT was 68 ms and Sti‐RVAT was 110 ms. (H) The 12‐lead ECG and the EGM recorded by the LBBP lead (Selective LBBP): The paced QRSd was 119 ms while Sti‐LVAT was 63 ms. (I) Po_His_ obtained during the intrinsic rhythm of a narrow QRS with an HV interval of 40 ms. (J) No Po_His_ recorded during escape beat of an RBBB morphology. (K) Po_LBB_ obtained with the LBB potential to ventricle interval of 20 ms. Fluoroscopic images at RAO 30° of: (L) RVAP; (M) RVSP; (N) HBP; (O) LBBP; at LAO 35° of (P) LBBP (white arrow depicted the depth of LBBP lead inside the septum via angiography through the sheath). HBP, his bundle pacing; LAO, left anterior oblique; LBB, left bundle branch; LBBP, left bundle branch pacing; RAO, right anterior oblique; RVAP, right ventricular apex pacing; RVSP, right ventricular septum pacing; Sti‐LVAT, stimulus to left ventricular activation time.

Unipolar pacing at different sites, including right ventricular apex pacing (RVAP), right ventricular septum pacing (RVSP), HBP, LBBP, and unipolar pacing from the ring electrode of LBBP lead (RVSP_ring_) were successively performed in each patient (Figure 1A−P). HBP and LBBP were attempted according to the literature by the lead (Mode 3830 69 cm; Medtronic) together with the sheath (Mode C315His; Medtronic) during the implantation procedure.^17^, ^18^ Briefly, His bundle potential was mapped around the atrioventricular grove with the pacing lead connection to the electrophysiology (EP) recording system (GE CardioLab EP Recording System 2000 GE Inc.) through the sheath (Model 3830, C315His; Medtronic Inc.) at RAO 30°. The lead was then turned four to five times clockwise for fixation. Afterward, the paced 12‐lead ECG was the same as the intrinsic during unipolar pacing, which confirmed His bundle capture (Figure 1I−J). LBBP was performed according to the His bundle location at RAO 30°. The pacing lead was moved toward the ventricular side about 1 cm across the tricuspid along the line between His location and RV apex and was deeply screwed into the subendocardium of the left ventricular septum. The criteria of successful LBBP were as follows according to our previous studies: (1) paced QRS of an RBBB pattern; (2) confirmation of selective LBBP (paced QRS of a typical RBBB pattern and a discrete component between stimulus and ventricle activation in intracardiac electrogram); and (3) the stimulus to left ventricular activation time (Sti‐LVAT) (Figure 1), which is defined as the interval from the pacing stimulus to the peak of the R wave in lead V5, shortening abruptly by increasing output or remaining shortest and constant at the final depth. The lead depth inside the interventricular septum was measured via angiography through the sheath at LAO 35° (Figure 1P). During the procedure, the atrial lead placed at the right atrium appendage and ventricular lead at different pacing sites above were temporarily connected to the programmer (Medtronic 2290) with DDD mode, AV delay of 150 ms, and pacing output of 3.5 V/0.5 ms during unipolar configuration at different sites above in each patient.

### 2.3 Data collection

QRS duration was measured from the onset to the end of the QRS wave and was compared during the intrinsic rhythm and different pacing sites. Sti‐LVAT and pacing stimulus to right ventricular activation time (Sti‐RVAT) (Figure 1G), defined as the interval from the pacing stimulus to the peak of R′ wave in lead V1, were recorded and compared during pacing at different sites. The transthoracic echocardiogram was used during the procedure. Echocardiographic parameters, including LVEF, left ventricular end‐diastolic volume (LVEDV), left ventricular end‐systolic volume (LVESV), interventricular mechanical delay (IVMD), and standard deviation (SD) of the time‐to‐peak myocardial sustained velocity of 12 left ventricular segments (TS‐12‐SD), tricuspid annular plane systolic excursion (TAPSE), were measured after 10 min of pacing at each site with a washout period of 5 min. IVMD was determined as the delay between left and right ventricular pre‐ejection intervals by Doppler (Figure S1). For TS‐12‐SD, pulsed‐wave Doppler and tissue synchronization imaging (Figure S2) were used to measure the left ventricular synchrony. All echocardiograms of our study were assessed by two experienced echocardiographers blinded to our study design. The fluoroscopy time of positioning the ventricular lead at different sites was also collected and compared. After collecting these parameters, LBBP lead was finally implanted together with the atrial lead connected to the ventricular and atrial port of the generator, respectively. Sensed/paced AV delay of 150/180 ms with pacing output of 3.5 V/0.5 ms by unipolar configuration were set in each case.

### 2.4 Follow‐up

During follow‐up (1, 3, 6, 12, and 18 months postprocedure), QRS durations and echocardiographic parameters, including LVEF, LVEDV, and LVESV were collected and compared. The procedure‐related complications, including lead dislodgement, perforation, device, or lead infection, pericardial effusion, and thromboembolism were also collected.

### 2.5 Statistical analysis

Continuous variables were expressed as mean ± SD and paired Student's *t‐*test was used to compare the difference between baseline and 6‐month follow‐up in each group. Analysis of variance test was used to perform comparison among more than two groups and was followed by the least significant difference test for multiple comparisons. Categorical variables were presented as numbers (percentages) by using Pearson's *X*² test or Fisher's exact test. The correlations between electrophysiological characteristics and echocardiographic parameters were performed by Pearson's analysis. All analyses were performed by SPSS version 22.0 (SPSS, Inc.) and a two‐sided *p* < .05 was considered statistically significant.

## 3 RESULTS

### 3.1 Electromechanical parameters during pacing at different sites

**Figure 2:**
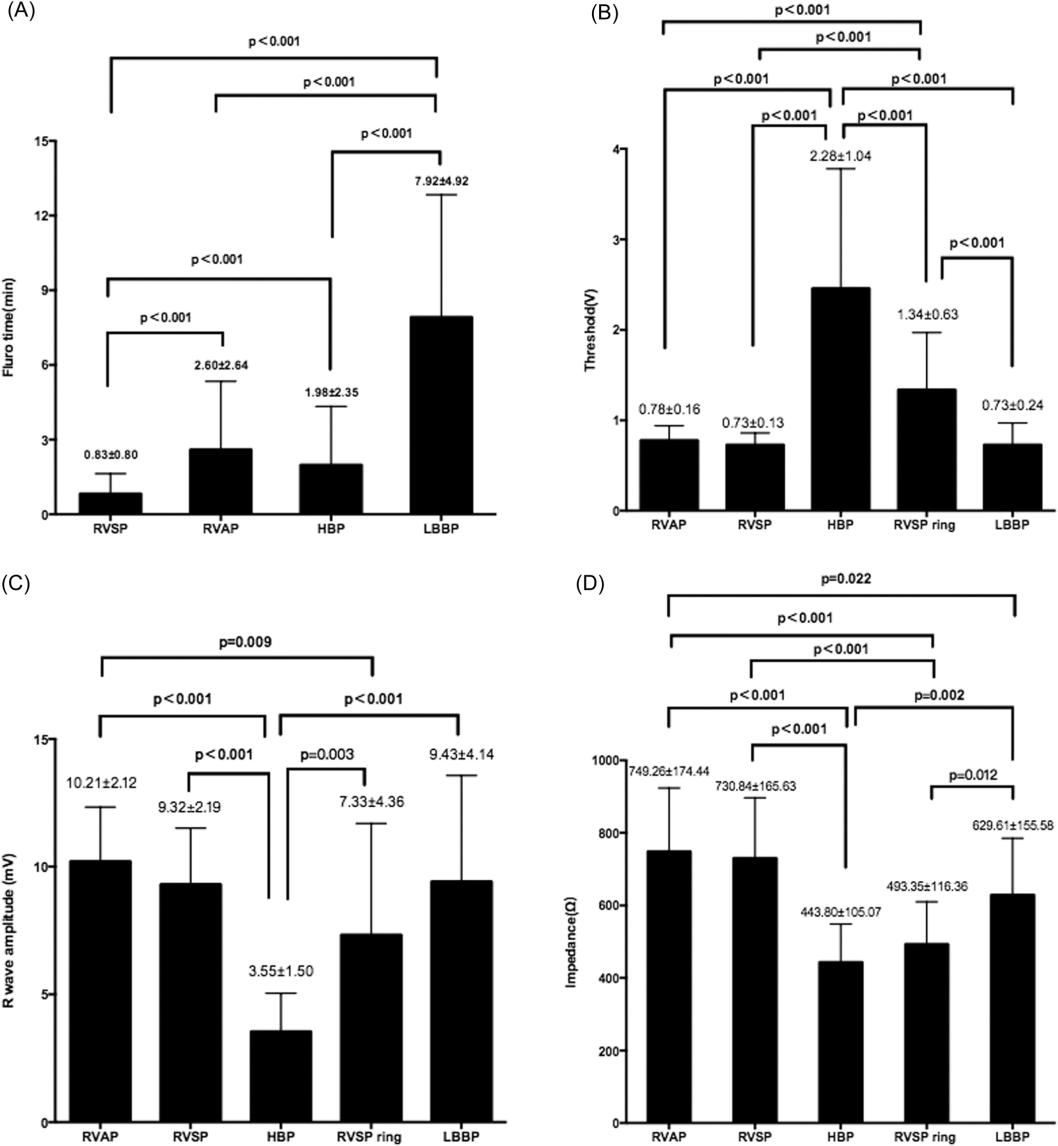
Fluoroscopic time and pacing parameters during pacing at different sites: (A) Fluoroscopic time; (B) Threshold; (C) R wave amplitude; (D) Impedance.

A total of 20 patients (mean age: 66.15 ± 13.65 years, 15 male) with complete AVB were enrolled and their baseline characteristics are shown in Table 1. Dual‐chamber pacemaker implantation with recordings of unipolar pacing at different sites was successfully achieved in all patients (Table 1) without procedure‐related complications. The mean depth of the lead was measured as 12.80 ± 0.89 mm into the interventricular septum. Pacing parameters during different pacing sites are shown in Figure 2B−D. The highest threshold (2.28 ± 1.04 V) and lowest R‐wave amplitude (3.55 ± 1.50 mV), as well as impedance (443.80 ± 105.07 Ω), were achieved in HBP among all the pacing sites (all *p* < .01). Threshold in LBBP (0.73 ± 0.24 V) was similar to RVAP (0.78 ± 0.16 V, *p* = .633) and RVSP (0.73 ± 0.13 V, *p* = .954) but significantly lower than RVSP_ring_ (1.34 ± 0.63 V, *p* < .001). For R‐wave amplitude, there was no difference between LBBP (9.43 ± 4.14 mV) and RVAP (10.21 ± 2.12 mV, *p* = .474) or RVSP (9.32 ± 2.19 mV, *p* = .914). With regard to impedance, LBBP (629.61 ± 155.58 Ω) is relatively lower than RVAP (749.26 ± 174.44 Ω, *p* = .022) but similar to RVSP (730.84 ± 165.63 Ω, *p* = .064). Moreover, higher impedance was demonstrated in LBBP when compared to RVSP_ring_ (493.35 ± 116.36 Ω, *p* = .012). As for LBBP, LBB potential was recorded in 15 cases (75%) and 16 cases (80%) achieved selective LBBP during the procedure.

**Table 1:** Baseline characteristics.

Variables	Results
Age (years)	66.15 ± 13.65
Male, *n* (%)	15 (75)
Body mass index (kg/m²)	24.58 ± 3.14
Hypertension, *n* (%)	10 (50)
Baseline electrocardiogram	
QRS durations (ms)	118.75 ± 24.63
Complete AVB, *n *(%)	20 (100)
Narrow QRS, *n* (%)	9 (45)
LBBB, *n* (%)	2 (10)
RBBB, *n* (%)	9 (45)
Baseline echocardiography	
Left atrium (mm)	40.30 ± 5.32
LVEDD (mm)	49.15 ± 5.46
LVEF (%)	62.12 ± 13.83
Ventricular septum (mm)	9.80 ± 1.58

Abbreviations: AVB, atrioventricular block; LBBB, left bundle branch block; LVEDD, left ventricular end‐diastolic diameter; LVEF, left ventricular ejection fraction; RBBB, right ventricular branch block.

### 3.2 Electrical and mechanical synchrony

**Figure 3:**
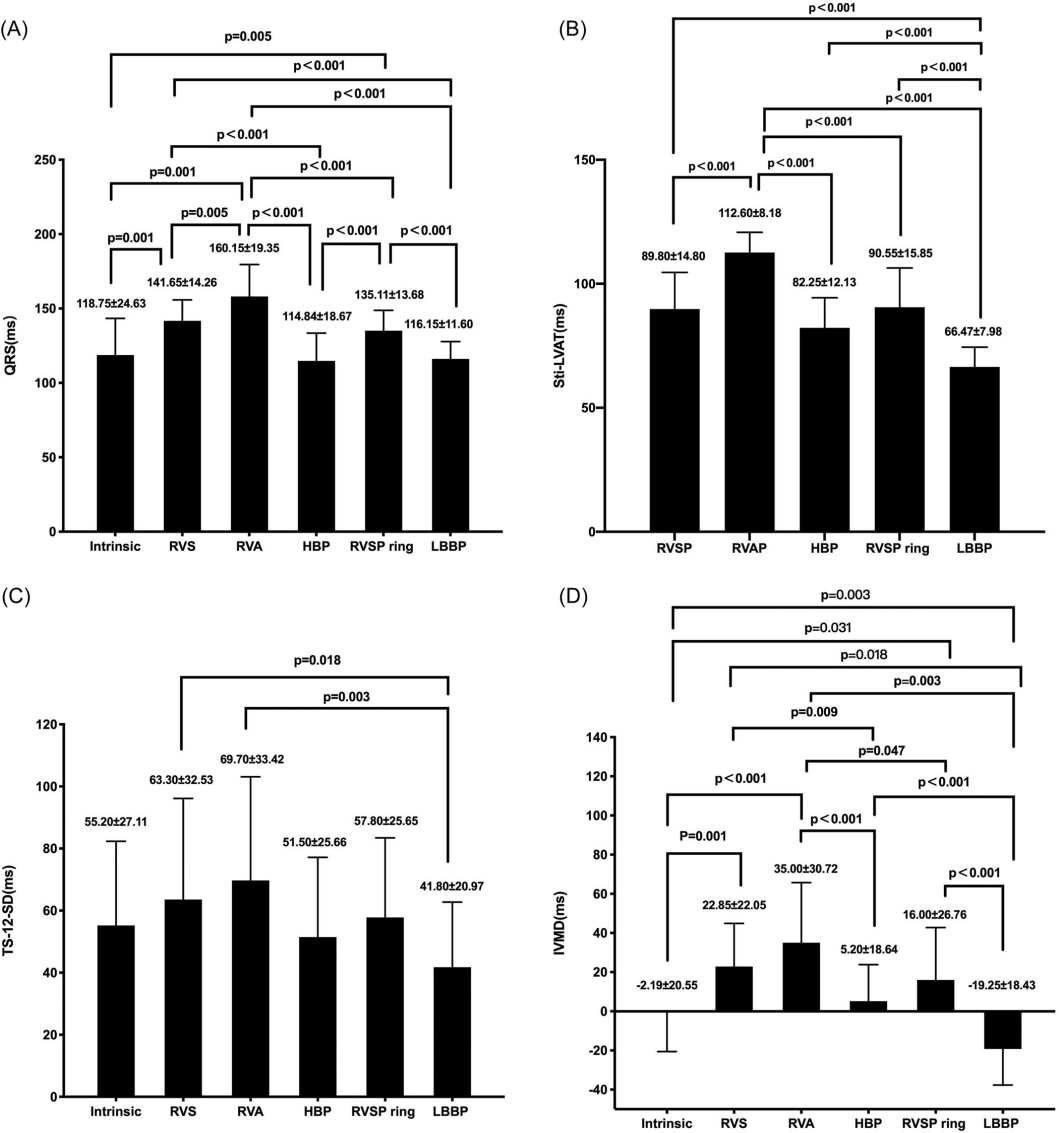
Electrical and mechanical synchrony during pacing at different sites: (A) QRSd; (B) Sti‐LVAT; (C) TS‐12‐SD; (D) IVMD. IVMD, interventricular mechanical delay; QRSd, QRS duration; Sti‐LVAT, stimulus to left ventricular activation time; TS‐12‐SD, standard deviation of the time‐to‐peak myocardial sustained systolic velocity of 12 left ventricular segments.

The electrocardiographic parameters during pacing at different sites are summarized in Table S1. RVSP (141.65 ± 14.26 ms, *p* = .001), RVAP (160.15 ± 19.35 ms, *p* = .001), as well as RVSP_ring_ (135.11 ± 13.68 ms, *p* = .005) resulted in a remarked increase in QRS duration compared to the baseline (118.75 ± 24.63 ms) (Figure 3A). QRS duration during HBP (114.84 ± 18.67 ms) and LBBP (116.15 ± 11.60 ms) was comparable to the intrinsic and hence, was both significantly narrower than RVSP, RVAP, and RVSP_ring_ (all *p* < .001). Concerning Sti‐LVAT, LBBP (65.47 ± 7.98 ms) showed the shortest Sti‐LVAT compared with RVSP (89.80 ± 14.80 ms, *p* < .001), RVAP (112.60 ± 8.18 ms, *p* < .001), HBP (82.25 ± 12.13 ms, *p* < .001), and RVSP_ring_ (90.55 ± 15.85 ms, *p* < .001) (Figure 3B).

Among all the different pacing sites, there was no significant difference in TS‐12‐SD as compared to baseline (55.20 ± 27.11 ms) while a decreasing trend could be seen during HBP (51.50 ± 25.66 ms) and LBBP (41.80 ± 20.97 ms) (Figure 3C). Whereas, TS‐12‐SD was significantly lower in LBBP compared with RVSP (*p* = .029) and RVA (*p* = .004). Significant negative means of IVMD was demonstrated in LBBP (−19.25 ± 18.43) than RVSP (22.85 ± 22.05 ms), RVAP (35.00 ± 30.72 ms), HBP (5.20 ± 18.64 ms), and RVSP_ring_ (16.00 ± 26.76 ms) (all *p* < .05) (Figure 3D). IVMD during HBP was similar to baseline and were significantly lower than RVSP (*p* = .009) and RVA (*p* < .001).

### 3.3 Evaluation of cardiac function during pacing at different sites

LVEF in HBP (62.71 ± 7.69%) and LBBP (62.93 ± 6.09%) was comparable to baseline (62.12 ± 13.83%) while a significantly decreased LVEF was identified in RVSP (59.40 ± 9.81%, *p* = .016), RVAP (60.47 ± 8.00%, *p* = .040), and RVSP_ring_ (58.50 ± 7.21%, *p* = .008) (Table S1). For other echocardiographic parameters, a trend toward decreased LVEDV and LVESV compared to baseline was indicated in HBP and LBBP but the difference did not reach statistical significance. TAPSE was significantly lower in RVSP (17.70 ± 3.06 mm, *p* = .004), RVAP (17.35 ± 2.82 mm, *p* = .001), and RVSP_ring_ (18.37 ± 2.81 mm, *p* = .033) compared with baseline (20.33 ± 2.54 mm) while HBP (19.53 ± 2.65 mm, *p* = .352) and LBBP (18.75 ± 2.65 mm, *p* = .069) remained stable.

### 3.4 Correlation between electrophysiological characteristics and echocardiographic parameters

**Figure 4:**
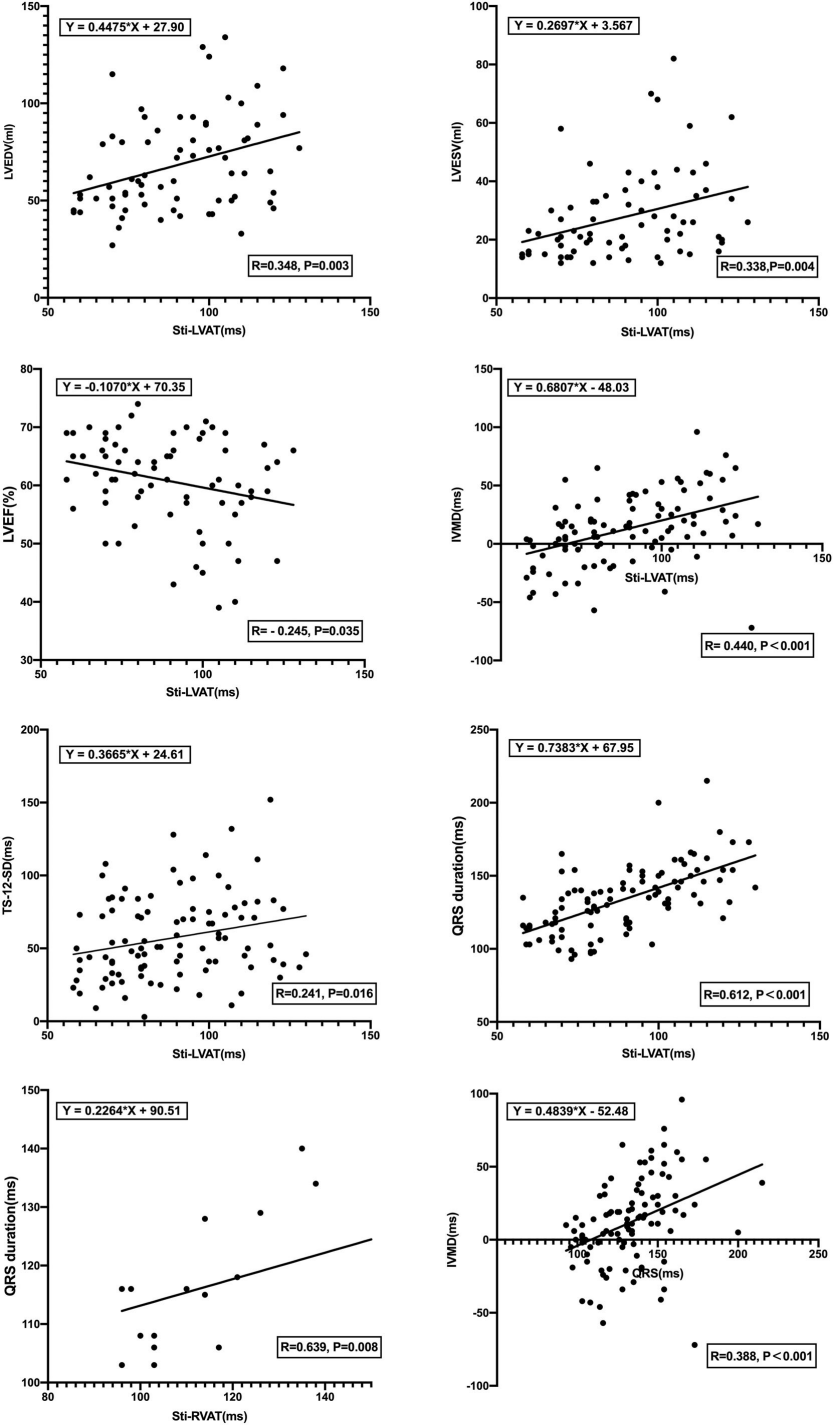
Linear correlations between electrical synchrony and echocardiographic parameters (A) Sti‐LVAT versus LVEDV; (2)Sti‐LVAT versus LVESV; (3) Sti‐LVAT versus LVEF; (4) Sti‐LVAT versus IVMD; (5) Sti‐LVAT versus TS‐12‐SD; (6) Sti‐LVAT versus QRS duration; (7) Sti‐RVAT versus QRS duration; (8) QRS duration versus IVMD. IVMD, interventricular mechanical delay; LVEF, left ventricular ejection fraction; LVEDV, left ventricular end‐diastolic volume; LVESV, left ventricular end‐systolic volume; Sti‐LVAT, stimulus to left ventricular activation time; TS‐12‐SD, standard deviation of the time‐to‐peak myocardial sustained systolic velocity of 12 left ventricular segments; Sti‐RVAT, stimulus to right ventricular activation time.

Correlations between electrophysiological characteristics and echocardiographic parameters are summarized in Table S3. Notable positive linear correlations could be observed between Sti‐LVAT and QRS duration (*r* = 0.612, *p* < .001), LVEDV (*r* = 0.348, *p* = .003), LVESV (*r* = 0.338, *p* = .004), TS‐12‐SD (0.241, *p* = .016), and IVMD (*r* = 0.440, *p* < .001), while a negative relationship between Sti‐LVAT and LVEF (*r* = −0.245, *p* = .035) was demonstrated (Figure 4A−F). However, QRS duration was not significantly correlated to the echocardiographic parameters except for IVMD (*r* = 0.388, *p* < .001). No significant association between Sti‐RVAT and echocardiographic parameters were confirmed, either.

### 3.5 Pacing parameters and echocardiographic outcomes at follow‐up

Follow‐up echocardiograms were obtained at least 18 months after the initial pacemaker implant in each patient. Stability was maintained in LBBP capture (0.81 ± 0.23 V, *p* = .300) and sensed R‐wave amplitude (10.98 ± 4.56 mV, *p* = .295) during unipolar configuration (Table S2). For unipolar pacing impedance, a significant decrease was found 6 months (510.61 ± 88.29 Ω, *p* = .008) after implantation and it was maintained till 18 months follow‐up (455.78 ± 73.68 Ω, *p* < .001) (Table S2). Neither LVEF (62.80 ± 6.01%, *p* = .952) nor QRS duration (113.75 ± 11.36 ms, *p* = .512) showed significant difference during follow‐up and none of the complications, including lead dislodgement, perforation, device or lead infection, pericardial effusion, or thromboembolism were reported.

## 4 DISCUSSION

The present study directly compared electrophysiological characteristics and echocardiographic parameters at different pacing sites in each patient during the procedure and the main findings were as follows: (1) similar to HBP, LBBP preserved better electrical and LV mechanical synchrony compared with conventional RV pacing (RVAP or RVSP); (2) our research provided the initial evidence of earlier LV electrical activation than RV during LBBP in accordance with interventricular synchrony (negative values of IVMD in LBBP); (3) there were significant correlations between Sti‐LVAT and echocardiographic parameters, including LVEDV, LVESV, LVEF, TS‐12‐SD, and IVMD.

### 4.1 Electrical synchrony

Paced QRS duration is demonstrated to be narrower in LBBP^12^, ^17^ and left ventricular septal pacing^19^ as compared to RVP. Wide QRS duration might be associated with ventricular dysynchrony and heart failure.^20^ During HBP and LBBP, the heart was activated fast through the conduction system, showing a significantly narrower QRS duration than RVAP or RVSP. When the comparison between HBP and LBBP, HBP showed a narrower QRS duration similar to the intrinsic than LBBP, and LBBP performed an RBBB pattern of paced QRS. Moreover, Sti‐LVAT, which is often used to reflect the lateral precordial myocardium depolarization time, was significantly decreased in HBP and LBBP than RVAP or RVSP. These findings demonstrated that HBP was the most physiological pacing strategy, which activated both ventricles fast through His bundle (LBB and RBB) while LBBP preserved physiological LV activation before RV through LBB. These findings were consistent with the literatures^18^: LBBP showed significantly reduced QRS duration and sti‐LVAT than RVSP in different individuals while our study further demonstrated it in each ventricular pacing dependent patient suffering from complete AVB during the implantation procedure.

A novel pacing site of the present study was RVSP_ring_, a specific RVSP during unipolar pacing from the ring electrode of LBBP lead. It might be a better pacing site than conventional RVAP or RVSP since the mean QRS duration during RVSP_ring_ was significantly decreased than RVAP and the trend was confirmed when comparing with RVSP but no statistical significance was found. Since the length between the tip and the ring of the lead (Mode 3830) was 10.8 mm, the pacing site of RVSP_ring_ was still RVS in most cases and might be a little bit deeper than conventional RVSP when the lead screwed deep inside the septum. Hence, the mean QRS duration and sti‐LVAT during RVSP_ring_ were significantly increased than LBBP, though they showed a decreased trend than RVAP or RVSP. And the pacing parameters were good as well during RVSP_ring_. Dual cathodal lead might be designed in the future to give an additional pacing option.

### 4.2 Mechanical synchrony

Better LV mechanical synchrony has been previously confirmed during LBBP than RVSP using SPECT MPI and echocardiogram.^15^, ^16^ The latter study by Cai et al.^15^ demonstrated that LV mechanical synchrony during LBBP was similar to that of native‐conduction concerning LV systolic dyssynchrony index and the SD of TS in the 12 segments, and the LV synchrony in LBBP was superior to the RVSP significantly in sick sinus syndrome patients. Consistently, our results showed the similarity of TS‐12‐SD between HBP, LBBP, and the intrinsic while increased TS‐12‐SD during RVP indicated LV dysynchrony, showing preserved LV mechanical synchrony in HPCSP. Whereas, the biggest advantage of the present intro‐patient‐controlled study in AVB was that the baseline difference between individuals had been minimized to the utmost extent to facilitate the measurement and comparison of the echocardiographic results at different pacing sites. However, there was no significant difference concerning TS‐12‐SD between LBBP and RVSP_ring_. It might be attributed to the study population (AVB patients without heart failure) and small‐sample‐sized study design.

Furthermore, IVMD was measured and compared in the study and showed a significant difference between LBBP and HBP or RVAP or RVSP or RVSP_ring_. IVMD evaluates the mechanical synchrony between LV and RV and is recognized as a predictive factor in CRT response.^21^ During LBBP, earlier LV activation than RV was confirmed by pacing activating LV before RV through LBB, showing an RBBB morphology of paced QRS. Consequently, significant negative means of IVMD during LBBP were confirmed as compared to positive ones at other pacing sites, indicating mechanical contraction of LV earlier than RV. Earlier LV electrical activation than RV during LBBP as an RBBB paced morphology in accordance with interventricular synchrony (shown as negative values of IVMD) was initially demonstrated in our research.

Concerning our findings of TS‐12‐SD and IVMD, HBP maintained inter and intraventricular synchrony while LBBP preserved LV synchrony with delayed RV activation. Conversely, RVP resulted in earlier RV activation and deteriorated LV synchrony.

### 4.3 Correlations between electrophysiological characteristics and echocardiographic parameters

To maintain satisfied heart function and mechanical synchrony after implantation, it is of significant importance to confirm a simple and reliable electrophysiological value correlated to echocardiographic parameters during pacing. Sti‐LVAT is indicated for the depolarization duration of the LV wall. Hence, a shorter Sti‐LVAT may represent rapid propagation of LV activation leading to synchronous LV contraction. During LBBP, Sti‐LVAT has been reported to be a useful parameter to determine LBB capture according to electrophysiological mapping while QRS duration failed to act as an ideal diagnostic value for LBB capture due to its delayed RV activation.^22^ As a main and novel finding, our research further confirmed that Sti‐LVAT was also notably correlated with LV systolic function (LVEDV, LVESV, and LVEF) and mechanical synchrony (TS‐12‐SD and IVMD), while QRS duration failed except for IVMD, indicating that Sti‐LVAT might be a better variable correlated to LV‐related echocardiographic parameters than QRS duration. Since a positive relationship was also shown between Sti‐LVAT and QRS duration, shorter Sti‐LVAT could be reasonably proposed to depict both favorable electrical synchrony and LV systolic function and mechanical synchrony. As for RV function, no remarkable correlations were shown in our study between Sti‐RVAT and TAPSE. Since our study was performed based on small sample size and merely TAPSE was included as RV‐related echocardiographic parameters, the correlation between Sti‐RVAT and RV function might be highly underestimated. In the future, further analysis of RV function (RV fractional area change, Tei index, speckle tracking derived free wall strain, and so forth.) was warranted to specifically evaluate the correlation with Sti‐RVAT during LBBP.

### 4.4 Limitations

The study focused on the electrical and mechanical synchrony at different pacing sites during the procedure. These findings were acute hemodynamic results. Thus, the long‐term hemodynamic effects of LBBP remain uncertain. In addition, it is a single‐center, self‐controlled observational study, with relatively small sample size. The main study population was AVB with narrow QRS or RBBB and normal LVEF. Consequently, the results of the study could not be generalized to patients with IVCD or heart dysfunction.

## 5 CONCLUSIONS

HPCSP provided better electrical and mechanical left ventricular synchrony than conventional RVP. While interventricular synchrony during LBBP was significantly different as compared to HBP, RVAP, and RVSP, showing earlier LV activation than RV, which was consistent with the RBBB pattern of paced QRS during LBBP. Sti‐LVAT might be a good electrophysiological parameter correlated to LV systolic function and mechanical synchrony.

## AUTHOR CONTRIBUTIONS

All authors have read and approved the manuscript. Methodology and manuscript writing: Xueying Chen, Qinchun Jin, Yanan Wang, Xiaolan Zhou. Data collection and pacing perfomance: Yufei Chen, Jingfeng Wang, Shengmei Qin, Jin Bai, Wei Wang, Yixiu Liang. Conceptualization and supervision: Haiyan Chen, Yangang Su, Junbo Ge.

## CONFLICTS OF INTEREST

The authors declare no conflicts of interest.

## Supporting information

Supporting information.

Supporting information.

Supporting information.

## Data Availability

The datasets generated and/or analysed during the current study are not publicly available duebut are available from the corresponding author on reasonable request.
