# Supplementary figures and images for: Evaluation of electrophysiological characteristics and ventricular synchrony: An intrapatient‐controlled study during His‐Purkinje conduction system pacing versus right ventricular pacing
Source: Clin Cardiol. 2022 May 3;45(7):723–32. doi: 10.1002/clc.23837 (PMC9286324; doi:10.1002/clc.23837)

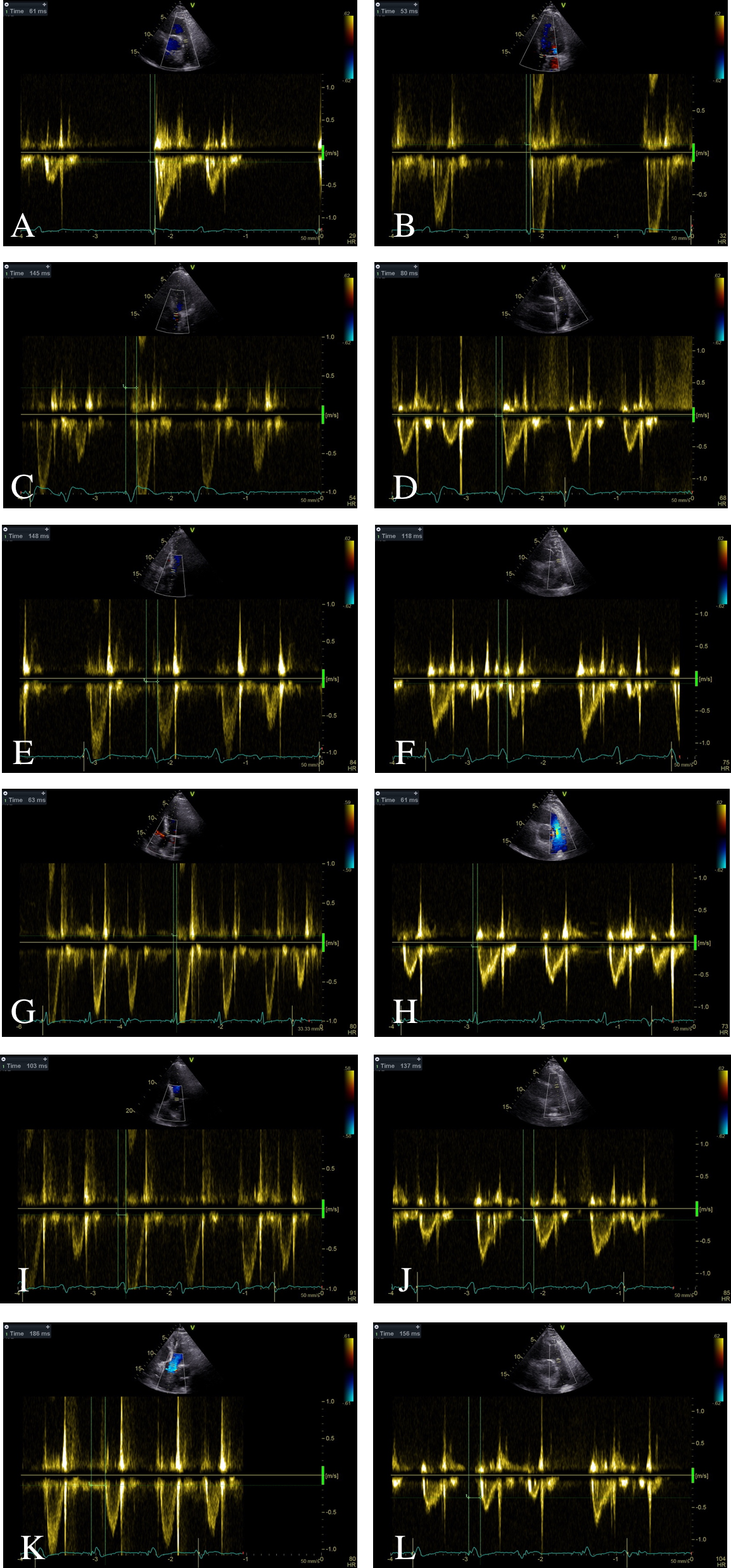

Supplement: Supplementary file 1 — Supporting information. [file CLC-45-723-s003.jpeg]

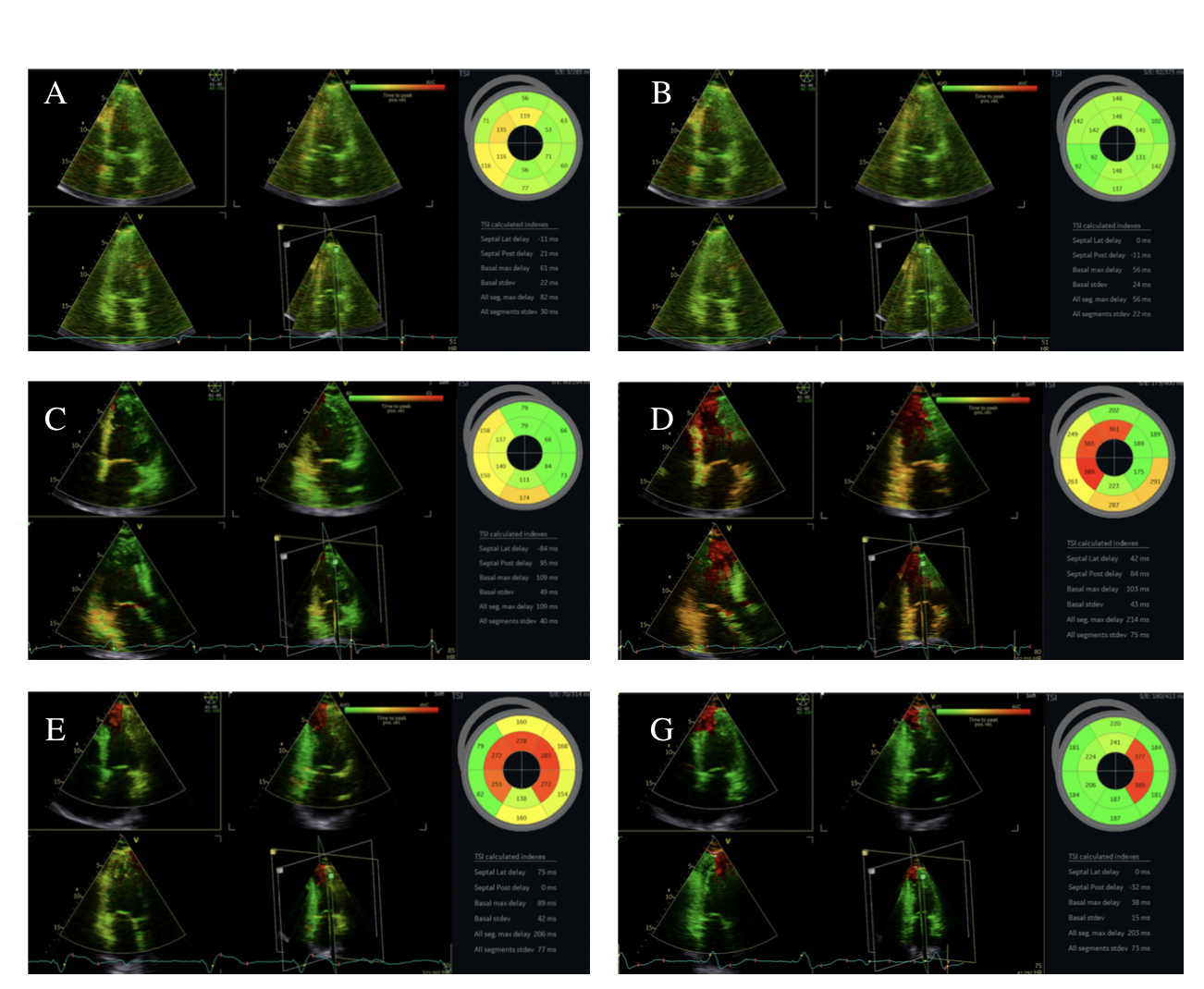

Supplement: Supplementary file 2 — Supporting information. [file CLC-45-723-s001.jpeg]
