# Supplementary material for: Evaluation of electrophysiological characteristics and ventricular synchrony: An intrapatient‐controlled study during His‐Purkinje conduction system pacing versus right ventricular pacing
Source: Clin Cardiol. 2022 May 3;45(7):723–32. doi: 10.1002/clc.23837 (PMC9286324; doi:10.1002/clc.23837)
Supplement: Supplementary file 3 — Supporting information. [file CLC-45-723-s002.docx]

**Supplementary table 1 Electrocardiographic and echocardiographic parameters**

**during pacing at different sites**

| Variables | Different pacing sites | | | | | | P# | | | |
| --- | --- | --- | --- | --- | --- | --- | --- | --- | --- | --- |
|  | Intrinsic | RVSP | RVAP | HBP | RVSP_ring_ | LBBP | LBBP vs RVSP | LBBP vs RVAP | LBBP vs HBP | LBBP vs RVSP_ring_ |
| Fluoro time(min) | - | 0.83±0.80 | 2.60±2.64 | 1.98±2.35 | - | 7.92±4.92 | ＜0.001 | ＜0.001 | ＜0.001 | - |
| QRSd(ms) | 118.75±24.63 | 141.65±14.26* | 160.15±19.35* | 114.84±18.67 | 135.11±13.68* | 116.15±11.60 | ＜0.001 | ＜0.001 | 0．821 | ＜0.001 |
| Sti-LVAT(ms) | - | 89.80±14.80 | 112.60±8.18 | 82.25±12.13 | 90.55±15.85 | 66.47±7.87 | ＜0.001 | ＜0.001 | 0.003 | ＜0.001 |
| TS-12-SD(ms) | 55.20±27.11 | 63.30±32.53 | 69.70±33.42 | 51.50±25.67 | 57.80±25.65 | 41.80±20.97 | 0.018 | 0.003 | 0.283 | 0.198 |
| IVMD(ms) | -2.19±20.55 | 22.85±22.05* | 35.00±30.72* | 5.20±18.64 | 16.00±26.76* | -19.25±18.43* | ＜0.018 | 0.003 | ＜0.001 | ＜0.001 |
| LVEF(%) | 62.12±13.83 | 59.40±9.81* | 60.47±8.00* | 62.71±7.69 | 58.50±7.21* | 62.93±6.09 | 0.218 | 0.388 | 0.940 | 0.129 |
| LVEDV(mL) | 79.87±23.17 | 69.93±27.62 | 70.00±26.43 | 67.57±23.59 | 65.57±23.59 | 66.33±21.10 | 0.689 | 0.604 | 0.886 | 0.934 |
| LVESV(mL) | 28.07±11.63 | 29.60±17.91 | 29.40±16.04 | 26.71±15.19 | 27.79±14.78 | 25.20±12.47 | 0.418 | 0.440 | 0.784 | 0.640 |
| TAPSE(mm) | 20.33±2.54 | 17.70±3.06* | 17.35±2.82* | 19.53±2.65* | 18.37±2.81 | 18.75±2.65 | 0.233 | 0.112 | 0.383 | 0.668 |

TS-12-SD: standard deviation of the time-to-peak myocardial sustained systolic velocity of 12 left ventricular segments; IVMD: interventricular mechanical delay; LVEF: left ventricular ejection fraction; LVEDV: left ventricular end-diastolic volume, LVESV: left ventricular end-systolic volume; RVAP: right ventricular apex pacing; RVSP : right ventricular septum pacing, HBP: His Bundle pacing, LBBP: left bundle branch pacing; RVSP_ring_: RVSP during unipolar pacing from the ring electrode of LBBP lead.

*: P value＜0.05 versus Intrinsic parameters

**Supplementary table 2 Pacing parameters by unipolar configuration for LBBP during 18 months follow-up:**

|  | Threshold  (V) | R wave amplitude  (mV) | Unipolar Impedance  (Ω) |
| --- | --- | --- | --- |
| Baseline | 0.73±0.24 | 9.75±3.58 | 629.61±155.48 |
| 1-month | 0.73±0.25 | 10.00±3.75 | 584.39±137.96 |
| 3-month | 0.72±0.22 | 10.48±3.75 | 548.67±100.51 |
| 6-month | 0.77±0.23 | 10.72±4.14 | 510.61±88.29** |
| 12-month | 0.76±0.20 | 10.99±4.02 | 479.22±83.90*** |
| 18-month | 0.81±0.23 | 10.98±4.56 | 455.78±73.68*** |

**: P＜0.01 versus baseline; ***: P＜0.001 versus baseline

**Supplementary table 3 Correlation between electrical synchrony and echocardiographic parameters**

|  | Pearson correlation | P value |
| --- | --- | --- |
| Sti-LVAT vs IVMD | 0.440 | **＜0.001** |
| Sti-LVAT vs TS-12-SD | 0.241 | **0.016** |
| Sti-LVAT vs LVEF | -0.245 | **0.035** |
| Sti-LVAT vs QRSd | 0.612 | **＜0.001** |
| Sti-LVAT vs LVEDV | 0.348 | **0.003** |
| Sti-LVAT vs LVESV | 0.338 | **0.004** |
| Sti-LVAT vs TAPSE | -0.134 | 0.189 |
| QRS vs IVMD | 0.388 | **＜0.001** |
| QRS vs TS-12-SD | 0.177 | 0.081 |
| QRS vs LVEF | -0.144 | 0.219 |
| QRS vs LVEDV | 0.136 | 0.252 |
| QRS vs LVESV | 0.149 | 0.208 |
| QRS vs TAPSE | 0.073 | 0.759 |
| Sti-RVAT vs IVMD | -0.077 | 0.778 |
| Sti-RVAT vs TS-12-SD | -0.178 | 0.509 |
| Sti-RVAT vs LVEF | 0.344 | 0.229 |
| Sti-RVAT vs QRSd | 0.639 | **0.008** |
| Sti-RVAT vs LVEDV | -0.101 | 0.731 |
| Sti-RVAT vs LVESV | -0.157 | 0.592 |
| Sti-LVAT vs TAPSE | -0.125 | 0.646 |

Abbreviations as in Supplementary table 1.
